# Peer Observation of Teaching Program for the Busy Hospitalist

**DOI:** 10.7759/cureus.26512

**Published:** 2022-07-02

**Authors:** Matthew Shaines, Todd Cassese

**Affiliations:** 1 Hospital Medicine, Albert Einstein College of Medicine, Bronx, USA

**Keywords:** teaching program, hospitalists, hospital based medicine, faculty development in medical education, peer observation

## Abstract

Background: Peer observation of teaching (POT) is a well-documented faculty development tool, but published research focuses mostly on programs in which participating physicians had protected time in their schedule in which to complete observations. Most programs nationally depend on hospitalists who have minimal, if any, protected time in their schedule and thus in order to complete these observations, will need to figure out a way to schedule these into their regular working day*.*

Objective: We determined whether a POT program, focused on hospitalists with minimal non-clinical time, scheduled around clinical responsibilities, could be feasible and acceptable.

Methods: Seven hospitalists participated in a POT pilot program from January 2019 to June 2019. Each hospitalist completed three 30-minute observations during this interval. At the completion of the pilot, the hospitalists were surveyed on their experience and results were assessed via open-ended narrative questions, which underwent thematic analysis.

Results: Twenty of the 21 scheduled observations (95%) were completed. Of the completed observations, 100% were completed while the observer was assigned to concomitant clinical duties. The survey response rate was 100%. From free-text responses, the following themes emerged: (1) Acceptable time commitment with minor inconvenience, (2) learning through observing and reflecting for personal growth, and (3) stress-free peer observations.

Conclusions: This study demonstrates the successful development and implementation of a POT program that served as a faculty development initiative focused primarily on hospitalists with minimal protected time.

## Introduction

Peer observation of teaching (POT) is a well-documented faculty development tool for educators, medical and non-medical alike [1-5]. While there is increasing research on peer observations amongst physician educators, there is a paucity of research that focuses on inpatient attending rounds specifically [6-8]. Reports to date examine mostly physicians in academic medical centers (AMC) who used non-clinical time in which to observe peers and provide feedback [6-8]. Upon a review of their methods and through discussions with the authors, peer observations were done infrequently, but were lengthy, at least an hour for each observation. However, many AMCs depend on physicians who often have minimal non-clinical time in which to participate in faculty development and thus we hypothesized that shorter observations, scheduled more frequently would be more acceptable to our faculty.

Some institutions use POT to evaluate, formatively or summatively, the teaching skills of their faculty. Our aim was to avoid the stigma, time commitment and faculty development needed for a robust evaluative process by creating a POT program focused on the reflective practice of the faculty observer. The emphasis on the observer has been reported by Beckman, who found that direct observation of Internal Medicine rounds ranked among his most meaningful educational experiences [3] and was the basis for the POT program described by Finn et al. in their 2011 work on a collaborative peer observation process [7]. Additionally, Hendry and Oliver, working outside of academic medicine, suggest that learning from watching a colleague teach may be more beneficial than receiving high-quality feedback [1].

We aimed to build upon Finn’s previous work on POT, by creating a pilot program that was practical for hospitalists working in a high-volume clinical setting. Our goal was to create a program in which faculty with contemporaneous clinical duties engaged in meaningful learning through observation and to assess the feasibility and acceptability of this approach.

This work has previously been accepted as an abstract for the Society of Hospital Medicine’s 2020 Virtual Competition.

## Materials and methods

Seven hospitalists were invited to participate in a six-month POT pilot from January 2019 to June 2019 at a large urban AMC. Each participant provided written consent prior to participating in the pilot. Invited participants were chosen in part because of their interest and/or expertise in teaching, but also to ensure the group was varied in terms of years of practice and quantity of teaching responsibilities (Table 1). Each hospitalist completed three observations over the six-month period. Observations were limited to only the other hospitalists in the pilot and were scheduled centrally by the hospitalist office to ensure each hospitalist had a variety of observations. Reminders were sent out via text message on the day prior to the scheduled observation. The focus of the observation was on teaching rounds, which take place between 8:30-10:30am on weekdays and are composed of one hospitalist, one resident, two interns and one to three medical students. Hospitalists could be scheduled to observe during any of their regularly scheduled activities (e.g., direct patient care, medicine consult, or administrative time), with the exception of if they themselves were scheduled for teaching rounds. To minimize conflicts with patient care, participants were asked to observe for 30 minutes sessions. Participants were informally surveyed prior to the start of the program, and this time commitment was thought to be acceptable. Prior to the first observation, all participants received a program overview document by email and then attended a 30-minute meeting to discuss goals and logistics for the program. In addition, participants reviewed the results of the Journal of Hospital Medicine’s 2017 article entitled “Techniques and Behaviors Associated with Exemplary Inpatient General Medicine Teaching” [8] to gain insights into what research has shown to be characteristics of high-quality educators.

**Table 1:** Demographic data of seven participants

Demographic data of 7 Participants			
Gender	Male	Female	
	5	3	
Yrs since residency graduation	<2	2-5	>5
	2	3	3
Months/year on teaching service	<2	2-4	>4
	2	3	3

In keeping with a previously published model by Finn et al. [7], rather than focusing on delivering and receiving feedback, the emphasis of the program was on observation and reflection so participants could develop new teaching strategies. Observers were encouraged to take notes during the observation session and were instructed to focus on teaching strategies observed rather than specific patient care practices, the content taught, and/or medical decision-making. The observer was asked to identify practices demonstrated by the observed hospitalist that they would like to incorporate into their future ward teaching rounds. Lastly, the observer was asked to email a one- to five-sentence summary of the teaching styles observed to the PI. These summaries were redacted of all identifying patient information and identifying observer and observee data. The redacted forms were collated into a single summary document to create a database of teaching styles which was shared with the members of the Division of Hospital Medicine.

After the completion of the six-month pilot, all participants were asked to complete an anonymous survey via Survey Monkey^TM^ answering 10 open-ended narrative questions pertaining to their experiences with the pilot. (see appendix for a complete survey)

Data from the free-text responses were subjected to thematic analysis to identify emerging themes. Coding was completed by three independent analysts, chosen from among the hospitalist participants. Subsequently, the two authors (MS, and TC) worked collaboratively to unify coding and identify themes over several iterative discussions. The study was reviewed and approved by Albert Einstein College of Medicine’s Institutional Review Board (number 050815).

## Results

Of the 21 scheduled observations, 20 (95%) were completed. The one incomplete observation was due to an urgent clinical issue that arose and was not able to be rescheduled due to logistical issues. Of the completed observations, 100% were completed while the observer was assigned to a clinical rotation: 15 while the observer was working on a non-teaching service (seeing on average 15-16 patients a day) and five while the observer was the supervising attending on the resident medicine consult rotation. The consult rotation requires the attending to be available for morning rounds from 9-11am to discuss new consults, review old consults and provide a brief didactic curriculum. The expectation is that attending will see all new consults, as well as select, follow-up consults while also being responsible for review and acceptance of transfer requests both from within the institution and externally (Table 2).

**Table 2:** Breakdown of hospitalist clinical assignment when completing their observation

Peer Observations		
Scheduled	21	
Completed	20	95%
Non-teaching service	15	75%
Medicine consult service	5	25%

All seven participants (100%) completed the survey. None of the participants raised any concerns about the pilot. Three major themes were identified: (1) Acceptable time commitment with minor inconvenience, (2) learning through observing and reflecting for personal growth, and (3) stress-free peer observations. See Table 3 for representative quotes.

**Table 3:** Thematic analysis of survey questions

Themes	Representative Quotes
Acceptable time commitment with minor inconvenience	"It was a little inconvenient to give up a half hour in the morning while on direct care. But since we only did a few throughout the year it did not require a major time commitment"
	"Just the right amount of time"
	"Can be challenging to step off the unit for 30 minutes to observe some mornings"
Learning through observing and reflecting for personal growth	"It was really helpful to see how others run rounds and learn from some of their techniques"
	"Great opportunity to learn from others and also acknowledge some of my own limitations"
	"I found it very helpful to see other attending rounds and plan to incorporate some of what I saw into my own teaching rounds"
	"I will definitely use some of my peers' techniques to engage the residents as much as I can during rounds"
	"Helps maintain innovation and interest in teaching"
Stress free peer observations	"[Felt] comfortable [with being observed]. If the everyone in the pilot understands the nature of the exercise it should in fact be a stress-free exercise."
	"No problem [having my teaching observed]."

Full survey questions can be found in the appendix. While we were initially interested in seeing if this pilot would affect stress or burnout, the results from this question were too limited to present at this time.

Acceptable time commitment with minor inconvenience

The initial question on the survey asked “What are your thoughts about the time commitment needed to participate in the teaching observation pilot?” 

Overall, the respondents believed the time commitment was reasonable, even when on a busy clinical service. One respondent did note that setting aside 30 minutes on a busy clinical service could be challenging, the infrequency of the observations made it more tolerable. 

Learning through observing and reflecting for personal growth

The questions representing this section were (1) “What feelings did you have about observing your colleagues’ teaching?”, (2) “What feelings did you have about having your teaching observed?” (3) “What did you get out of participating in this pilot?” and (4) “How do you think this pilot will impact your future teaching (if at all)?”

Respondents overall enjoyed observing their peers and were not overly stressed or intimidated. A majority of the responses believed theirs was value in the experience and would utilize the observations to improve upon their own teaching in the future.

Stress-free peer observations

We asked our participants (1) “What concerns do you have about the pilot?” as well as (2) How did this pilot influence your level of stress/burnout? None of our participants raised any concerns nor any negative affect on their stress level.

## Discussion

As fewer resources are being allocated for clinical teaching, AMCs are turning to hospitalists to teach the next generation of residents, all the while having limited or no protected time in which to develop their teaching skills. It is thus essential that we look to develop programs that can improve the faculty development of teaching inpatient medicine, during a busy clinical day. The results of this pilot study indicate that hospitalists, including ones with minimal non-clinical time and limited teaching responsibilities, found a POT program with a relatively brief time commitment, based on learning through observation, not only acceptable and feasible but enjoyable.

Previous research on POT programs, often reports on observations lasting at least an hour in duration. We chose a reduced time of 30 minutes, to minimize interference with patient care duties. To offset this decreased observation time, we scheduled our observations more frequently (three times over six months) than most other previously reported programs. In addition, we chose not to focus on providing feedback on teaching given concerns of increasing the time commitment for already busy clinicians. While lack of a feedback process may seem to be a flaw in a faculty development initiative, previously published work has identified the act of observing and reflecting as possibly being one of the most, if not, the most valuable experience in the POT process [1,3,9,10].

Based on our survey data, the majority of the responses indicated that the time commitment was reasonable, and no one noted that it negatively impacted their clinical work. None of the hospitalists noted concerns with watching their colleagues and more importantly with their rounds being observed. In addition, the majority of participants stated that observing their colleagues was a positive experience that will lead to changes made in their teaching practice in the future.

We believe this is the first report of a POT program that incorporated observations into a busy clinical schedule with observers assigned concomitant clinical duties. Previously published studies on POT during ward rounds focused primarily on academic clinician-educators, with observations scheduled during the non-clinical time, which would not be possible for our cohort of physicians and most teaching hospitalist programs nationally [7,11,12].

The study findings are limited by the small sample size of hospitalists. As this pilot selected highly motivated hospitalists, there was likely bias introduced as to their attitudes toward both the feasibility and acceptability of the process. Given these seven hospitalists were pre-selected by the Division to participate, they possibly represented a sample of higher functioning clinicians, in which 30 minutes away from their clinical duties was more manageable than for more inexperienced hospitalists. In addition, the study was conducted over a relatively short period of time. As the program expands and continues, there may potentially be issues of sustainability. Despite pausing the program during the two surges of COVID-19 in our area, our aim for this academic year is to expand the program to include over 40 hospitalists spread over three campuses and evaluate its feasibility and acceptability.

## Conclusions

POT is a well-documented faculty development tool, but previously only evaluated its implementation among faculty who utilized their protected, non-clinical time in which to participate in observations and feedback. As residency programs continue to depend on clinicians, who have ever-increasing clinical demands in their schedule, to teach the next generation of physicians, there is an urgent need to develop creative faculty development solutions that can be implemented within the constraints of a busy clinical day. Responding to this need, we were able to successfully develop and implement a POT pilot program that served as a faculty development initiative that focused on hospitalists with minimal non-clinical time. Our physicians found this program acceptable from a time-commitment standpoint while remaining educationally valuable. We believe our POT program is thus more generalizable to the majority of the teaching hospitalist programs nationally.

## Appendices

Post POT Survey

What are your thoughts about the time commitment needed to participate in the teaching observation pilot?

What feelings do you have about observing your colleagues’ teaching?

What feelings do you have about having your teaching observed?

What did you get out of participating in this pilot?

How do you think this pilot will impact your future teaching (if at all)?

What concerns do you have about the pilot?

How did this pilot influence your level of stress/burnout?

What aspects of the program would you suggest we continue in future iterations?

What aspects of the program should we modify to make it a better experience in future iterations?

What additional training do you think the Division of Hospital Medicine should create to help you improve as a teacher?
